# Comparison of six machine learning methods for differentiating benign and malignant thyroid nodules using ultrasonographic characteristics

**DOI:** 10.1186/s12880-023-01117-z

**Published:** 2023-10-12

**Authors:** Jianguang Liang, Tiantian Pang, Weixiang Liu, Xiaogang Li, Leidan Huang, Xuehao Gong, Xianfen Diao

**Affiliations:** 1 School of Pharmacy & School of Biological and Food Engineering, Changzhou University, Changzhou, Jiangsu 213164 China; 2 Health Science Center, Shenzhen University, Shenzhen, 518060 China; 3 School of Biomedical Engineering, Shenzhen University, Shenzhen, 518060 China; 4 Guangdong Key Laboratory for Biomedical Measurements and Ultrasound Imaging, Shenzhen, 518060 China; 5 National-Regional Key Technology Engineering Laboratory for Medical Ultrasound, Shenzhen, 518060 China; 6 College of Computer Science and Technology, Jilin University, Changchun, 130012 China; 7 Guangzhou Medical University, Guangzhou, 510182 China; 8 Department of Ultrasound, First Affiliated Hospital of Shenzhen University, Second People’s Hospital of Shenzhen, Shenzhen, 518035 China

**Keywords:** Machine learning, Support vector machine, Logistic regression, Linear discriminant analysis, Random forest, GlmNet, K-nearest neighbors, Thyroid nodule, Paired t-test

## Abstract

**Background:**

Several machine learning (ML) classifiers for thyroid nodule diagnosis have been compared in terms of their accuracy, sensitivity, specificity, negative predictive value (NPV), positive predictive value (PPV), and area under the receiver operating curve (AUC). A total of 525 patients with thyroid nodules (malignant, *n* = 228; benign, *n* = 297) underwent conventional ultrasonography, strain elastography, and contrast-enhanced ultrasound. Six algorithms were compared: support vector machine (SVM), linear discriminant analysis (LDA), random forest (RF), logistic regression (LG), GlmNet, and K-nearest neighbors (K-NN). The diagnostic performances of the 13 suspicious sonographic features for discriminating benign and malignant thyroid nodules were assessed using different ML algorithms. To compare these algorithms, a 10-fold cross-validation paired t-test was applied to the algorithm performance differences.

**Results:**

The logistic regression algorithm had better diagnostic performance than the other ML algorithms. However, it was only slightly higher than those of GlmNet, LDA, and RF. The accuracy, sensitivity, specificity, NPV, PPV, and AUC obtained by running logistic regression were 86.48%, 83.33%, 88.89%, 87.42%, 85.20%, and 92.84%, respectively.

**Conclusions:**

The experimental results indicate that GlmNet, SVM, LDA, LG, K-NN, and RF exhibit slight differences in classification performance.

## Background

There is a high incidence of thyroid nodules following the widespread use of high-resolution ultrasound in clinical practice. Ultrasonography plays an important role in the diagnosis of thyroid nodules because it is noninvasive, economical, and convenient. Most thyroid nodules are benign; however, it is difficult to differentiate malignant nodules from benign nodules owing to their hidden early clinical symptoms [1, 2]. Therefore, differentiating benign and malignant thyroid nodules is challenging. Known suspicious US features of differentiated thyroid nodules are margins, borders, calcification, and shape [3, 4]. In this paper, we chose 13 features, including conventional US features, and features based new imaging techniques, such as strain elastosonography (SE) and contrast-enhanced ultrasound (CEUS); see more details in the Materials section.

Machine learning (ML) is one of the fastest developing fields in the computer science field. ML serves as a useful reference tool for classification following the development of artificial intelligence.

Several types of classifiers are used in ML. The support vector machine (SVM), random forest (RF), logistic regression, GlmNet, linear discriminant analysis (LDA), and *K*-NN are the most common classifiers.

The original SVM was proposed by Vapnik and Ya in 1963. The current standard originated in 1993 and was proposed by Corte and Vapnikdition. SVM is a core machine-learning technology for resolving a variety of classification and regression problems, which produces nonlinear boundaries by constructing a linear boundary in a large, transformed version of the feature space [5]. SVM has been applied to all types of problems, such as object and handwritten digit recognition and image and text classification. The general form of the decision function *f* (*x*) for SVM:

1 $$f (x) =\sum\nolimits_{i=1}^{n}{a}_{i}{y}_{i}k(x, {x}_{i}) + b$$

where *k*(*x, x*_*i*_) is the kernel function, *b* is the bias, 0 ≤ *α*_*i*_ ≤ *C* andΣ(*α*_*i*_*y*_*i*_) = 0.where *α*_*i*_ can be obtained through training, and *C* is a penalty term parameter set by user [5–7]. In this study, the Gaussian kernel function ${k}_{\gamma }(X, X^{\prime}) = e(-\hspace{0.17em}\gamma ||X\hspace{0.17em}-\hspace{0.17em}X^{\prime}{||}^{2})$ was used to address the nonlinearity classification [5]. The SVM with a Gaussian kernel is implemented in MATLAB using the LIBSVM toolkit, which is a library for SVMs and is publicly available.

**Fig. 1:**
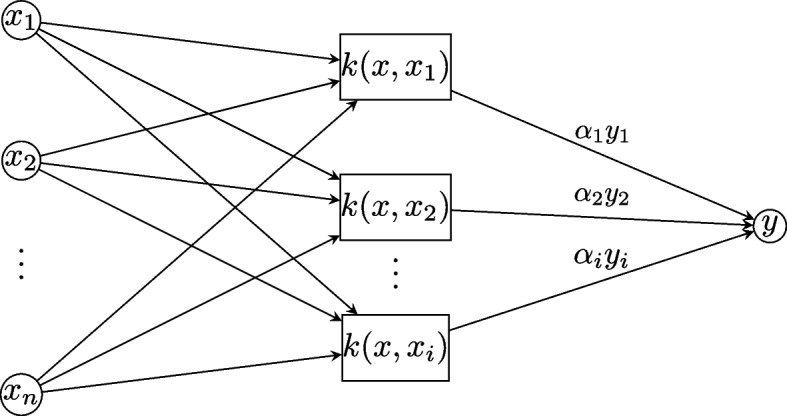
The architecture of the support vector machine (SVM)

Figure 1 is the architecture of an SVM. *x* = [*x*_1_*,* *x*_2_*,… x*_*n*_] is an *n*-dimensional input feature vector, and *y* is the decision value.

2 $$y=sgn\left(\sum\nolimits_{i=1}^{n}{a}_{i}{y}_{i}k\left(x,{x}_{i}\right)+b\right)$$

RFs were first proposed by Breiman and Cutler. RF is a versatile machine-learning algorithm that can implement regression, classification, and dimensionality reduction. Random forests are a combination of decision trees, where each decision tree depends on the values of a random vector sampled independently [8]. The performance of random forests is quite similar to that of the bootstrap aggregating algorithm for many problems, which depends on the strength of the individual trees in the forest and the correlation between trees [5]. The steps of the algorithm are as follows:

- *N* samples are randomly sampled with replacements from the data set.
- The* m* features are randomly sampled from all the features. A certain strategy (*CART*) is used to select one feature from *m* features as the split attribute of the node.
- The above two steps are repeated *n* times, that is, to generate *n* decision trees to form a random forest.
- After each decision, the final vote is confirmed as the category for new data.

*K−*Nearest Neighbors is memory-based and requires no preprocessing of the sample and no model to fit [5, 9]. Given point *x*_0_, *k* points that are the closest distance to *x*_0_ were found. The majority vote is then used to classify *k* points [5]. The decision rule is defined as follows.

3 $$\widehat{f}(X)=\frac{1}{k}\sum\nolimits_{{x}_{i}\in N_k (\mathbf{X})} {y}_{i}$$

where *N*_*k*_(**X**) is the neighborhood of **X**.

Logistic regression is a generalized linear regression model and is the most common algorithm used in binary classification problems. The decision function of the logistic regression is

4 $$Z=sigmoid\left({\theta }^{T}x\right)=\frac{1}{1+{e}^{{-\theta }^{T}x}}$$

where *sigmoid* (*.*) is the activation function and *x* is the matrix of the input data. The value is set to 1 if *Z* ≥ 0.5. By contrast, the value is regarded as zero if *Z* < 0.5.

The GlmNet is a generalized linear model with penalized maximum likelihood. GlmNet solves the following binomial likelihood function:

5 $${\mathrm{min}}_{{\beta }_{0},\beta }\left\{-\frac{1}{N}\sum\nolimits_{i=1}^{N}\left[{y}_{i}\left({\beta }_{0}+{x}_{i}^{T}\beta \right)+\mathrm{log}\left(1+{e}^{{\beta }_{0}+{x}_{i}^{T}\beta }\right)\right]+{\lambda P}_{a}\left(\beta \right)\right\}$$

where

$${P}_{a}\left(\beta \right)=\left(1-\alpha \right)\frac{1}{2}{\Vert \beta \Vert }_{{l}_{2}}^{2}+\alpha {\Vert \beta \Vert }_{{l}_{1}}$$

where *α* is the mixing factor, *λ* is the regularization parameter, *and P*_*α*_(***β***) is the elastic net penalty. The model is a ridge regression model when *α* is zero. The model is a lasso regression when *α* = 1.

In the space of dimensionality reduction and data classification, LDA is wildly used. The principle of LDA is to project the labeled data into a lower-dimensional space using the projection method; therefore, the projected points can be easily distinguished, and the points of the same category will be closer to the projected space. The principle of LDA is to maximize the distance between classes and and to minimize the distance between the within-class [10]. The mapping function is

6 $$Y = {W}^{T} XI$$

where *X* is the dataset to be categorized. The original central point of Category *i* is

7 $${m}_{i}=\frac{1}{n}\sum\nolimits_{x\epsilon {D}_{i}}x$$

where *D*_*i*_ represents the set of points belonging to category *i* and *n* is the number of *D*_*i*_.

The variance before the projection of category *i* s

8 $${S}_{i}^{2}=\sum\nolimits_{x\epsilon {D}_{i}}(x - {m}_{i}){(x - {m}_{i})}^{T}$$

The central point after the projection of category *i* is:

9 $${\widehat{m}}_{i}={W}^{T}{m}_{i}$$

The variance after the projection of Category *i* is

10 $$\begin{array}{l}{\widehat{S}}_{i}^{2}=\sum\limits_{y\epsilon {Y}_{i}}{\left(y-{\widehat{m}}_{i}\right)}^{2}\\ =\sum\limits_{x\epsilon {D}_{i}}{\left({W}^{T}x-{W}^{T}{m}_{i}\right)}^{2}\\ \begin{array}{l}=\sum\limits_{x\epsilon {D}_{i}}{W}^{T}\left(x-{m}_{i}\right){\left(x-{m}_{i}\right)}^{T}W\\ ={W}^{T}{S}_{i}^{2}W\end{array}\end{array}$$

where *Y*_*i*_ is the data set after *D*_*i*_ mapping.

Assuming that there are two categories in the dataset, the loss function is

11 $$\begin{array}{l} J\left(W\right)=\frac{{(\widehat{{m}_{1}}-\widehat{{m}_{2}})}^{2}}{\widehat{{S}_{1}^{2}}+\widehat{{S}_{2}^{2}}}\\ =\frac{{\left({W}^{T}{m}_{1}-{W}^{T}{m}_{2}\right)}^{2}}{{W}^{T}{S}_{1}^{2}W+{W}^{T}{S}_{2}^{2}W}\\ \begin{array}{l} =\frac{{W}^{T}{\left({m}_{1}-{m}_{2}\right)}^{2}W}{{W}^{T}{\left({S}_{1}^{2}+{S}_{2}^{2}\right)}W}\\ =\frac{{W}^{T}{S}_{B}^{2}W}{{W}^{T}{S}_{W}^{2}W}\end{array}\end{array}$$

where $${S}_{B}^{2}={({m}_{1}-{m}_{2})}^{2} and\, {S}_{w}^{2}={S}_{1}^{2}+{S }_{2}^{2}$$  

The goal is to find the *W* that makes *J*(*W*) the biggest.

The motivation behind this study is to develop a better understanding of the classification process and evaluate it in terms of accuracy and sensitivity, specificity, NPV, PPV, and AUC, and to analyze the weaknesses and strengths of known classifiers in differentiating malignant from benign nodules. These issues are important and valuable for the application of machine classifiers in thyroid research and for clinicians and researchers who would like to gain an understanding of the classification process and analysis.

## Results

The performance of these classifiers is summarized in Table 1. Based on the results in Table 1, logistic regression works relatively well and achieves maximum accuracy (86.48%), which shows the best classification performance. However, there are only slight differences in the performances of the six classifiers.

**Table 1:** Six evaluate performances for different classifiers

Classifier	Accuracy	Sensitivity	Specificity	NPV	PPV	AUC
SVM	85.14%	79.39%	89.56%	84.98%	85.38%	85.11%
RF	85.14%	82.46%	87.21%	86.62%	83.19%	91.38%
GlmNet	86.29%	82.46%	89.23%	86.89%	85.45%	92.6%
LDA	86.48%	81.14%	90.57%	86.22%	86.85%	92.51%
LG	86.48%	83.33%	88.89%	87.42%	85.20%	92.84%
*K*-NN	84.95%	74.56%	92.93%	82.63%	89.01%	—

A statistical test method was applied to classifier performance differences to quantitatively compare the classifiers [11]. The 10-folder cross-validation paired t-test was applied to compare the two classifiers, and the significance level was 0.05. When the *p*-value was < 0.05, the two classifiers were significantly different. Table 2 shows the *p*-values of the paired t-tests. The results indicate that the six classifiers have no significant differences.

**Table 2:** The result of paired t-test of classifier differences

Classifier	SVM	RF	GlmNet	LDA	LG	k-NN
SVM	—	0.9854	0.5846	0.4216	0.0656	0.9044
RF		—	0.2720	0.1916	0.4442	0.8695
GlmNet			—	0.8646	0.9147	0.2218
LDA				—	0.9804	0.1394
LG					—	0.3485
k-NN						—

## Discussion

In this analysis, the cross-validation technique and paired t-test method were applied to tune parameters and assess classifier performance differences, respectively. The experimental results indicate that GlmNet, SVM, LDA, logistic regression, *K*-NN, and random forests exhibit slight differences in classification performance. The reason for this result may originate from our data, as all variables and labels are binary.

For clinical research, there are lots of classifiers for a real application. It is useful for clinician to select an optimal classifier. Our exprehensive comparison study may be such an effort for helping clinicians in their real problem.

## Conclusions

The strength of this study is that 13 features regarding gender, SE, and CEUS in combination with other 10 conventional US features were used to compare different classifiers in the diagnosis of malignancy and benign disease. This study had a few limitations. First, the sample size was small. Moreover, this was a retrospective study. The established model requires further research to validate and support it. Large-sample studies are expected to be performed in the future. Second, the data in this study were binary. Finally, it is a good way to use other model data with new methods such as deep learning for thyroid nodule diagnosis [12, 13].

## Materials and method

### Materials

A database of 525 patients (396 females and 129 males) who underwent conventional US, SE, and CEUS at Shenzhen Second People’s Hospital was retrospectively reviewed. The patients were subdivided into two groups based on the final pathology results: those with benign thyroid nodules (*n* = 297) and those with malignancy (*n* = 228). We chose 13 features based on our clinical experience and data as many as possible according to our current imaging equipment; all features are listed in Table 3. In this study, 10 conventional US features of malignancy were: irregular margins, ill-defined borders, taller-than-wide shapes, hypoechogenicity or marked hypoechogenicity, microcalcification, posterior echo attenuation, peripheral acoustic halo, interrupted thyroid capsule, central vascularity, and suspected cervical lymph node metastasis. We chose the images according to clinicical experience.

**Table 3:** The used 13 features for comparison

Variable	Name/Description
X1	Gender/Sex
X2	irregular margins
X3	ill-defined borders
X4	taller-than-wide shape
X5	hypoechogenicity or marked hypoechogenicity
X6	microcalcification
X7	posterior echo attenuation
X8	a peripheral acoustic halo
X9	an interrupted thyroid capsule
X10	central vascularity
X11	suspected cervical lymph node metastasis
X12	strain elastosonography
X13	contrast-enhanced ultrasound

SE is an advanced technology used to evaluate tissue elasticity through the action of an external force. Under the same conditions, soft materials are more distorted than hard materials [2]. The degree of distortion under an external force was used to evaluate tissue hardness. Based on the fact that benign thyroid nodules are softer than malignant nodules, SE is used to differentiate benign from malignant nodules [2].

The SE score was based on Xu’s scoring system [14] as follows: Score 1: the nodule is predominantly white; Score 2: the nodule is predominantly white with few black portions; Score 3: the nodule is equally white and black; Score 4: the nodule is predominantly black with a few white spots; Score 5: nodules are almost completely black; and Score 6: nodules are completely black without white spots. A nodule was considered malignant if the score was greater than 4. CEUS is a new technique that infuses microbubbles into blood capillaries, which are smaller than the erythrocytes. Owing to the ultrasound scattering effect produced by blood capillaries, it can estimate the blood perfusion features of thyroid nodules to evaluate angiogenesis [2].

By comparing the echogenicity brightness between the thyroid nodule and surrounding parenchyma at peak enhancement, the degree of enhancement was classified as hypo, iso, hyper, or no enhancement. According to the echogenicity intensity of the thyroid nodules, the enhancement identity was classified as homogeneous and heterogeneous. Additionally, the nodule was regarded as malignant if the pattern of enhancement was heterogeneous hypoenhancement.

### Method

All statistical analysis in this study was conducted using MATLAB software, version R2015a.

Different classifiers had different tuning parameters. There were no tunable parameters for the LDA and logistic regression classifiers. There were two parameters for RF.

The number of randomly selected variables *m* and decision trees *ntree* was fixed at 500 as the default value for the two tunable parameters. Therefore, RF was the only tunable parameter in this study. The tunable parameter of *K*-NN is the number of neighbors *K*. The other classifiers had two tunable parameters (SVM and GlmNet). The SVM had two tunable parameters: the Gaussian kernel(*γ*) and penalty coefficient (*c*). There were two tunable parameters for GlmNet: the mixing factor (*α*) and the regularization parameter (*λ*).

In this study, a five-fold cross-validation technique was used to tune the parameters for the classifiers. In each folder, based on a grid of parameter values, the optimal tunable parameters of the classifier were determined using five-fold cross-validation of the training data, which maximized classification accuracy. Table 4 provides a grid of parameter values from which the optimal parameters of the classifiers are chosen by five-fold cross-validation of the training data. This study evaluated performance using 10-folder cross-validation, including sensitivity, specificity, accuracy, PPV, NPV, and AUC.

**Table 4:** A grid of parameter values for different classifiers

Classifier	Parameter Values
SVM	*c ∈ {− *8: 0*.*8: 8*}*; *g ∈ {− *8: 0*.*8: 8*}*
RF	*m ∈ {*1: 1: 13*}*; *ntree* = 500
GlmNet	*α ∈ {*0*.*1: 0*.*1: 1*}*; *λ ∈ {*2*.*0789*e − *4*,* 3*.*6329*e − *4*,* 6*.*3485*e − *04*,* 0*.*0011*,* 0*.*0019*,* 0*.*0034*,* 0*.*059*,* 0*.*0103*}*
*K*-NN	*k ∈ {*2: 2: 22*}*

## Data Availability

The datasets used and analysed during the current study are available from the corresponding author on reasonable request.

## Abbreviations

ML: Machine learning
US: Conventional ultrasonography
SE: Strain elastosonography
CEUS: Contrast-enhanced ultrasound
SVM: Support vector machine
LDA: Linear discriminant analysis
RF: Random forest
LG: Logistic regression
KNN: K-nearest neighbors
NPV: Negative predictive value
PPV: Positive predictive value
AUC: The area under the receiver operating curve

## References

[1] Batawil N, Alkordy T (2014). Ultrasonographic features associated with malignancy in cytologically indeterminate thyroid nodules. Eur J Surg Oncol.

[2] Pang T, Huang L, Deng Y, Wang T, Chen S, Gong X, Liu W (2017). Logistic regression analysis of conventional ultrasonography, strain elastosonography, and contrast-enhanced ultrasound characteristics for the differentiation of benign and malignant thyroid nodules. PLoS One.

[3] Zhao RN, Zhang B, Yang X, Jiang YX, Lai XJ, Zhang XY (2015). Logistic regression analysis of contrast-enhanced ultrasound and conventional ultrasound characteristics of sub-centimeter thyroid nodules. Ultrasound Med Biol.

[4] Chng CL, Kurzawinski TR, Beale T (2015). Value of sonographic features in predicting malignancy in thyroid nodules diagnosed as follicular neoplasm on cytology. Clin Endocrinol.

[5] Franklin J (2010). The elements of statistical learning: data mining, inference and prediction. Publ Am Stat Assoc.

[6] Drucker H, Burges CJC, Kaufman L, Smola AJ, Vapnik V (1997). Support vector regression machines. Adv Neural Inf Process Syst.

[7] Cortes C, Vapnik V (1995). Support-vector networks. Mach Learn.

[8] Breiman L (2001). Random forests. Mach Learn.

[9] Cover T, Hart P (1967). Nearest neighbor pattern classification. IEEE Trans Inf Theory.

[10] Hastie T, Tibshirani R, Friedman J. The Elements of Statistical Learning. New York: Springer; 2009.

[11] Hothorn T, Hornik K, Zeileis A (2006). Unbiased recursive partitioning: a conditional inference framework. J Comput Graph Stat.

[12] Zhu YC, AlZoubi A, Jassim S, Jiang Q, Zhang Y, Wang YB, Ye XD, Hongbo DU (2021). A generic deep learning framework to classify thyroid and breast lesions in ultrasound images. Ultrasonics.

[13] Zhu YC, Jin PF, Bao J, Jiang Q, Wang X (2021). Thyroid ultrasound image classification using a convolutional neural network. Ann Transl Med.

[14] Zhang YF, He Y, Xu HX, Xu XH, Liu C, Guo LH, Liu LN, Xu JM (2014). Virtual touch tissue imaging on acoustic radiation force impulse elastography. J Ultrasound Med.

